# Review, role of lactoferrin in preventing preterm delivery

**DOI:** 10.1007/s10534-022-00471-9

**Published:** 2022-12-10

**Authors:** Katsufumi Otsuki, Takshi Nishi, Tetsuro Kondo, Kazutoshi Okubo

**Affiliations:** Department of Obstetrics and Gynecology, Showa University Koto Toyosu Hospital, 5-1-38 Toyosu, Koto-Ku, Tokyo, 135-8577 Japan

**Keywords:** Preterm birth, *Lactobacillus* species, Lactoferrin, Bacterial vaginosis, Chorioamnionitis

## Abstract

Prevention of preterm birth (PTB) is a global challenge and is one of the most important issues to be addressed in perinatal care. The hypothesis that ascending lower genital infection leads to PTB has been tested in numerous in vitro and in vivo studies. For patients with intractable vaginitis or high-risk patients with successive PTBs, mainly due to intra-uterine infection, the vaginal flora is enhanced to increase systemic immunity and locally propagate *Lactobacillus* species. It has been shown that the administration of lactoferrin (LF), a prebiotic with minimum side effects, may be effective in suppressing PTB. This hypothesis has been evaluated in this review using various relevant test examples. The findings suggest that LF may play a role in inflammatory protection in pregnant human cervical tissue. The antibacterial and anti-cytokine effects of LF in human-derived mucus-producing cervical cell lines were also demonstrated. It was also clarified that LF suppresses PTB and improves the prognosis of pups in inflammation-induced PTB animal models. Thus, we have identified that LF, a prebiotic contained in breast milk, can be clinically applied to suppress PTB in humans and to prevent PTBs in high-risk pregnancies.

## Introduction

Prevention of preterm birth (PTB) is a global challenge and is one of the most important issues that should be addressed in perinatal care. The primary cause of neonatal mortality is infant immaturity (Bick 2012). The PTB rate varies globally and regionally (Bick 2012), being ≥ 10% in some developed countries. In the United States, the PTB rate has been declining significantly in recent years, but it is still high at just below 10% (Beck et al. 2010). In Japan, the PTB rate has been improving since 1990 and is now approximately 5.7%. Although the perinatal mortality rate in Japan is the lowest in the world (Organization 2017a, b) the mortality rate of preterm infants born before 30 weeks of gestation is high and accounts for approximately 75% of perinatal mortality, excluding congenital malformations. Even if they survive, preterm infants have various difficulties. The long-term prognosis of very low birth weight infants (weighing less than 1000 g) shows that more than 20% of them have issues with neurological development (Mathews and Driscoll 2017) (MacDorman et al. 2014). However, there are many concerns regarding PTBs that are still being investigated.

The hypothesis that ascending lower genital tract infections lead to PTB has been tested in numerous in vitro and in vivo studies (Keelan et al. 2003). Goldenberg (Taylor-Robinson and Furr 1997a, b) and Bennett et al. (Bennett and Elder 1992a, b) reported that commensal bacteria in the vagina do not produce prostaglandins independently. In contrast, the pathogenic organisms locally produce enzymes, such as sialidase and mucinase, decreasing the efficacy of the cervical mucus, which acts as a protective barrier against bacteria, thereby promoting the ascending invasion of bacteria (McGregor et al. 1994). The invasion of pathogenic bacteria induces the production of inflammatory cytokines from the lower reproductive organs into the decidua (Keelan et al. 2003), and it has been proved that cytokines induce the activation of the prostaglandin production system in the amniotic membrane, decidua, and uterine muscle as well as cause chorioamnionitis (CAM) (Goldenberg et al. 2000). This causes uterine muscle contraction and expansion, cervical contraction, membrane rupture, and bacterial invasion of the uterus. Cytokines also promote the production of matrix metalloproteinases (MMPs) in the villi and amniotic membranes. MMPs are also known to cause cervical ripening and amniotic membrane weakening. (Cox et al. 1988a, b) found that the bacterial endotoxin, lipopolysaccharide (LPS), found in amniotic fluid, stimulates the decidua and induces the production of cytokines and prostaglandins, leading to PTB. Romero, Cox, and others have detected endotoxins in amniotic fluid. Andrews et al. (Andrews et al. 1995) reported that spontaneous PTB cases have higher interleukin (IL)-6 levels in the amniotic fluid than induced PTB cases.

Moreover, the usefulness of probiotic therapy without using antibacterial agents (Mastromarino et al. 2013) and the usefulness of lactoferrin (LF)—a prebiotic—for refractory vaginal inflammation (Otsuki and Imai 2017) have been reported.

## Main text

### “Role of LF in preventing preterm delivery”

For patients with intractable vaginitis or high-risk patients with successive PTBs, mainly due to intra-uterine infection, the vaginal flora is enhanced to increase systemic immunity and locally propagate *Lactobacillus* species. It has been shown and verified that administration of LF, a prebiotic that rarely causes adverse effects, may be effective in suppressing PTB. This review evaluates the role of LF based on human cases and animal studies.

LF is a glycoprotein abundantly present in human milk and neutrophils, and exists as one of the prebiotics in the human body. It has been reported that LF has antibacterial and anti-inflammatory cytokine properties and does not suppress the growth of *Lactobacillus* species (Valenti eta al. 2018). Thus, the administration of LF as a preemptive medicine may potentially lead to the prevention of PTB. To test this hypothesis, the following studies (I–IV) were conducted.

Samples were collected from patients with the approval of the Institutional Review Board and the consent of the patients, and animal experiments were conducted under the approval of the Animal Care and Use Committees. Regarding human use, approvals were obtained from Medical Ethics Committees; oral and written explanations were given to patients and their families; and consent was obtained.

#### I. Examination of human LF dynamics in human cervical mucus and amniotic fluid (Table 1)

**Table 1:** Studies regarding the use of lactoferrin for preventing preterm delivery

Types of study	Models of study	Results
I. Examination of human LF dynamics in human cervical mucus and amniotic fluid		
(1) human LF (h-LF) in cervical mucus:	Cervical mucus from pregnant women	LF concentration was significantly low (p < 0.01) in women with cervicitis/CAM combined condition. No changes in h-LF levels were observed with respect to gestational age
(2) LF levels in amniotic fluid:	Amniotic fluid from pregnant women	The h-LF concentration increased with gestational age
II. Examination of anti-cytokine action and its mechanism of bovine LF in amnion cell culture system and antibacterial action and its mechanism of recombinant human LF in human cervical gland cell culture system:		
(1)Experimental system using amniotic membrane cell culture:	Primary culture from human amniotic membrane	The administration of 1000 ng/mL b-LF further suppressed cytokine production
(2) Cervical gland cell culture system:	Human cervical cell lines [HeLa (ATCC; CCL-2) (mucus-producing cells) and ME-180 (ATCC; HTB-33) (non-mucus-producing cells)],	rh-LF inhibited the growth of E. coli in the presence of human mucin-producing cervical cells
(3) Experimental system using macrophage culture:	Macrophage phagocytosis (ATCC; CRL-21192) of gram-negative bacteria [*E. coli* (0064, Ec5, RS128), *Salmonella* Minnesota (Re595)]	rh-LF did not show a clear opsonic effect on macrophage phagocytosis of gram-negative bacteria
III. Examination of PTB inhibitory effect of recombinant human LF in animal experimental system:		
(1) Experimental system using mouse PTB model	Mice (Female; C3H / HeNCrj, Male; B6D2F1)	The suppressive effect of rh-LF on PTB was evaluated
(2) Experimental system using rabbit cervicitis/PTB model (*E. coli* intracervical administration):	New Zealand White Rabbit	The suppressive effect of rh-LF on PTB was evaluated
(3) Experimental system using rabbit cervicitis /cervical ripening model (LPS vaginal administration):	New Zealand White Rabbit	Vaginal administration of rh-LF has potential as a local treatment to prevent cervical ripening
IV. The effect of prebiotic bovine LF on improving the vaginal flora and preventing PTB in humans		
Use of oral pb-LF tablets (700 mg/day)	Women (age, 30–39 years) with a history of several pregnancy losses or preterm delivery and refractory bacterial vaginosis	All cases were reported and improvement of the vaginal flora was observed; however, *Lactobacillus* species appeared or were predominant 1–3 months after initiating pb-LF administration in all the participants (except one where it appeared immediately after the start of pb-LF use). All cases were delivered after 35 weeks of gestation. In addition, no adverse events, which could be associated with the administration of pb-LF, were found in all the infants and patients who completed delivery

##### (1) Human LF (h-LF) in cervical mucus

The cervical mucus was collected from 652 participants using a cotton swab, and LF levels were determined by sandwich enzyme-linked immunosorbent assay **(**ELISA) using an anti-human LF immunoglobulin G polyclonal antibody (Otsuki K et al. 1997) (Sawada et al. 2006a, b). Differences based on conception, gestational age, and cervicitis/CAM complications were examined [CAM (−): n = 199, CAM ( +): n = 29, non-pregnant women: n = 37]. The h-LF concentrations observed were as follows: non-pregnant women, 4.6 ± 6.8 μg/mL; normal pregnant women, 11.5 ± 15.8 μg/mL; and cervicitis/CAM combined pregnant women, 2 ± 3.7 μg/mL. Thus, the h-LF concentration was significantly low (p < 0.01) in women with cervicitis/CAM. No changes in h-LF levels were observed with respect to gestational age.

##### (2) LF levels in amniotic fluid

Variations in **h**-LF and IL-6 levels with gestational age in the presence or absence of CAM were examined to determine the correlation between **h**-LF and IL-6 [CAM (−): n = 31, CAM( +): n = 28] (Otsuki et al. 1998, 1999). The **h**-LF concentration increased with gestational age. At delivery, **h-**LF concentration of the CAM ( +) cases was 8.76 ± 0.65 μg/mL, which was significantly (p < 0.01) higher than that in CAM (−) cases (0.86 ± 0.81 μg/mL). Thus, a positive correlation was found between h-LF and IL-6 (r = 0.91).

#### II. Examination of anti-cytokine action and its mechanism of bovine LF in amnion cell culture system and antibacterial action and its mechanism of recombinant LF in human cervical gland cell culture system

##### (1) Experimental system using amniotic membrane cell culture

Amniotic membrane was collected from a patient scheduled for caesarean section owing to an overdue pregnancy, and primary culture was performed. The addition of 100 ng/mL LPS induced inflammatory cytokine production, and the inhibitory effect of bovine LF (b-LF) (Morinaga Milk Industry, Zama, Japan) at concentrations of 100 and 1000 ng/mL on inflammatory cytokine production was investigated (Otsuki et al. 1998, 1999). LPS-induced IL-6 production was significantly suppressed (p < 0.05) by the administration of 100 ng/mL b-LF. The IL-6 levels observed in the control, LPS-administered, and b-LF-administered groups were 110 ± 20, 512 ± 15, and 214 ± 40 pg/mL/2 × 10^5^ cells, respectively. The administration of 1000 ng/mL b-LF further suppressed cytokine production.

##### (2) Cervical gland cell culture system

Using human cervical cell lines [HeLa (ATCC; CCL-2) (mucus-producing cells) and ME-180 (ATCC; HTB-33) (non-mucus-producing cells)], the antibacterial effect of recombinant human LF (rh-LF) (Aggenix Inc., Houston, TX, USA) on *Escherichia coli* was investigated by Brock (2002). rh-LF significantly suppressed the growth of *E. coli* in the presence of HeLa cells (mucus producing). However, no inhibitory effect was observed in the presence of ME-180 cells (non-mucus producing). This study thus demonstrated that rh-LF inhibits the growth of *E. coli* in the presence of human mucin-producing cervical cells. It has been suggested that mucus-producing cervical cells play an important role while the non-productive mucus cell do not.

##### (3) Experimental system using macrophage culture

The effect of opsonins on macrophagic phagocytosis (ATCC; CRL-21192) of gram-negative bacteria [*E. coli* (0064, Ec5, RS128) and *Salmonella* Minnesota (Re595)] was examined. We also investigated macrophage activation through the production of nitric oxide (NO) and tumor necrosis factor alpha (TNF-α) (Otsuki et al. 2002a, b). Although rh-LF did not show a clear opsonic effect on macrophage phagocytosis of gram-negative bacteria, it promoted NO and TNF-α production by macrophages (TNF-α: 1.8 ± 0.2 μM, Nitrite → 2.4 ± 0.1, NO: 14 ± 0.1 ng/mL → 29 ± 0.2).

#### III. Examination of PTB inhibitory effect of rh-LF in animal experimental system

##### (1) Experimental system using mouse PTB model

In the PTB model, LPS (50 μg/kg) was intraperitoneally administered to mice (Female; C3H/HeNCrj, Male; B6D2F1). rh-LF-treated mice were administered an intraperitoneal injection of either rh-LF dissolved in an isotonic sodium chloride solution (1 mg/200 µL) 1 h before each LPS injection (1:00 and 4:00 PM) or isotonic sodium chloride solution. (1) The inhibitory effect of rh-LF on PTB was then examined in various groups (Group A: saline administration (n = 12), Group B: saline + LPS administration. (n = 12), Group C: rh-LF + LPS administration group (n = 12), Group D: rh-LF + saline administration group (n = 4)). The delivery dates of mice in groups A, B, C, and D were 19.0 ± 0, 16.2 ± 0.4, 18.0 ± 0.8, and 19.0 ± 0 days, respectively. Thus, the gestation period was significantly extended (p < 0.05) compared to control group. (2) A similar treatment, as described above, was performed on separate groups of mice, and the concentrations of inflammatory cytokines (IL-6 and TNF-α) in maternal serum and amniotic fluid were examined [Group E (same as Group A) (n = 14), Group F (same as Group B) (n = 12), and Group G (same s Group C) (n = 7)]. IL-6 and TNF-α were quantified using ELISA. IL-6 concentrations in the maternal serum of groups E, F, and G were 497 ± 39, 1628 ± 115, and 244 ± 59 pg/mL, respectively. TNF-α concentrations in the maternal serum of groups E, F, and G were 0 ± 0, 113.7 ± 48.8, and 7.1 ± 30.2 pg/mL, respectively. Thus, levels of both cytokines were significantly lower (p < 0.05) in the rh-LF + LPS-administered group than in the saline + LPS-administered group. A similar tendency has been observed for cytokine concentrations in the amniotic fluid (Mitsuhashi et al. 2000; Sasaki et al. 2004a, b) (Otsuki et al. 2005).

##### (2) Experimental system using rabbit cervicitis/PTB model (E. coli intracervical administration)

New Zealand White Rabbits were used for this experiment. Under anesthesia, rh-LF (5 mg/body weight) was administered directly into the cervical canal using a histero fiberscope and a sterile catheter with diameters of 5 and 1.2 mm, respectively. Two hours later, *E. coli* (107 colony forming units/body weight) was administered at the same site and the following parameters were assessed: (1) duration of pregnancy, (2) cytokines in blood and amniotic fluid (cytotoxic assay method), (3) degree of CAM and pathological examination of inflammation, and (4) animal infant mortality (fetal + neonatal) and pathological examination. The animals were divided into the following groups: group A: saline administration (n = 6), group B: saline + *E. coli* administration (n = 8), group C: rh-LF + *E. coli* administration (n = 10) and group D: rh-LF + saline administration (n = 3) (Hasegawa et al. 2005a, b).

*Number of consecutive pregnancy days:* The numbers of continuous pregnancy days in the A, B, C, and D groups were 7.0 ± 0, 3.6 ± 1.3, 5.3 ± 2.1, and 6.0 ± 1 days, respectively. In groups B and C, the number of pregnancy days was significantly higher (p < 0.05) than that in groups A and D.

*Cytokines in blood and amniotic fluid:* Serum TNF-α concentrations at the time of delivery were 45.6 ± 10.2 pg/mL (Group A), 96.6 ± 22.6 pg/mL (Group B), and 69.2 ± 12.0 pg/mL (Group C). The serum TNF-α concentrations at the time of delivery had a tendency to decrease, although that in Group C was significantly different from that in Group B. The amniotic fluid TNF-α concentrations at the time of delivery were 218.7 ± 27.2 pg/mL (Group B) and 48.5 ± 24.7 pg/mL (Group C). The amniotic fluid TNF-α concentration at the time of delivery in Group C was significantly low compared with that in Group B.

*Degree and inflammation of CAM:* Group A (saline solution-inoculated) animals and Group C (rh-LF-treated) animals showed no inflammatory exudates or necrosis in the endometrium, decidua, or placenta. In contrast, Group B (no treatment) animals had diffuse infiltration of the decidua and sub-placental zone separation with polymorphonuclear leukocytes, consistent with histologic deciduitis. In the amniotic membrane and placenta, the infiltration of inflammatory cells was suppressed in the rh-LF + *E. coli*-administered group compared to that in the saline + *E. coli*-administered group, and the degree of CAM was mild.

*Animal infant mortality rate* The animal infant mortality rates in groups A, B, C, and D were 0, 42.3, 16.0, and 0%, respectively, which were significant in the rh-LF + *E. coli*-administered group compared to that in the saline + *E. coli*-administered group (p = 0.05).

##### (3) Experimental system using rabbit cervicitis/cervical ripening model (Vaginal LPS administration)

Using the same animal model as previously described, LPS and rh-LF levels, during the 3 days from the 14th to the 16th day of pregnancy, were determined. The animals were grouped based on the use of drug inserts, such as vaginal tablets [group A: suppository base only (n = 4), group B: rh-LF (10 mg) + LPS (100 ng) (n = 6), and group C: LPS (100 ng) (n = 6)]. Two days later, the cervical canal was removed under anesthesia, and the suppressive effect on cervical ripening (determined using techniques, such as extension test, pathological examination, and expression of MMPs using western blot) was examined (Yakuwa et al. 2007). The histological study showed remarkably loose and edematous connective tissue in Group B cervices. Cervical tissues in Group A were not different from those in Group C. Extension lengths were 2.2 ± 0.2 mm in Group A, 7.0 ± 2.7 mm in Group B, and 1.7 ± 0.3 mm in Group C.

These results suggest that rh-LF inhibits cervical maturation induced by LPS in a rabbit model and may have the potential to prevent preterm delivery caused by cervical infection and ripening.

The study also demonstrates that vaginal administration of rh-LF has the potential as a local treatment to prevent cervical ripening caused by LPS and suggests that this may be a defense mechanism against infection for preventing PTB in a rabbit model.

Regarding the depressive effect of LF on LPS-induced inflammatory cytokine production, it is speculated that LF directly neutralizes the LPS effect as well as causes an increase in the body’s overall immune function. Zhang (Zhang et al. 1999) and Miyazawa (Miyazawa et al. 1991) reported that LF neutralizes endotoxins in cases of septicemia and is possibly a curative treatment for septicemia by decreasing TNF-α concentration. LF, although thought to have various indirect actions (Shau et al. 1992) (Zimecki et al. 1998) (Lima and Kierszenbaum 1985, 1987), is expected to have a potential clinical application in the prevention of premature birth in the near future.

#### IV. The effect of LF in improving the vaginal flora and preventing PTB in humans

Based on the results of I–III, the target population for this study included patients with intractable bacterial vaginitis, which is a high-risk cause of PTB, and those who had endometritis or were unable to give birth to a live infant due to repeated late miscarriages and premature birth. Among these, the participants included in the study were those who underwent a vaginal discharge culture test but only showed the presence of group B *Streptococcus* and other bacterial species, but not *Lactobacillus* species (or *Lactobacillus* species were not predominant).

At the pre-pregnancy or post-pregnancy stage before cervical length shortening, the signs of imminent PTB became apparent in cases where the vaginal flora did not improve, despite the appropriate use of antibacterial agents and hormonal agents. Eventually, six patients were enrolled. Five of them (age, 30–39 years) had a history of several pregnancy losses or preterm delivery and refractory bacterial vaginosis and received prebiotic bovine LF (pb-LF) (Shimizu 2004; Ono et al. 2010) (NRL Pharma, Kawasaki, Japan) therapy delivered normally, and one of them was not pregnant. Two of the women started pb-LF therapy before becoming pregnant, and the others started therapy from 11 to 21 weeks of gestation when refractory bacterial vaginosis was diagnosed. All patients had high-risk factors for preterm delivery, including a history of delivery before 30 weeks of gestation. They also had abnormal bacterial flora and bacterial vaginosis. Vaginal *Lactobacillus* was either absent or very scarce before pb-LF administration. Therefore, we obtained ethical approval from our Institutional Review Board and written informed consent from each patient to administer vaginal suppositories (150 mg/day) in the evening after shower or bath and oral tablets (700 mg/day) after breakfast of pb-LF. Changes in the vaginal bacterial flora were confirmed after the initiating pb-LF administration, and its applicability in suppressing PTB was explored. It has been jointly researched at several facilities apart from our hospital (Otsuki et al. 2014) (Otsuki and Imai 2017) that by the end of the year 2017, all cases showed improvement of the vaginal flora; however, *Lactobacillus* species appeared or were predominant 1–3 months after initiating pb-LF administration in all the participants (except one where it appeared immediately after the start of LF use). All patients delivered after 35 weeks of gestation. In addition, no adverse events, which could be associated with the administration of pb-LF, were found in all the infants and patients who completed delivery.

## Discussion

Although there has been progress in obstetric management for preventing PTB as well as in neonatal management, prevention of PTB is a global challenge and is one of the most important issues that should be addressed in perinatal care.

Recent studies have indicated a relationship between PTB and intrauterine infection, particularly CAM, wherein the PTB rate has been 5.7%, and despite the control of various factors (such as maternal, fetal, genetic, endocrinal, immune, and nutritional factors), a downward trend has not been achieved (Organization 2017). CAM is induced by various types of microorganisms and bacterial vaginosis, which is considered as a precursor to this condition. In recent years, CAM has been posited as a cause of PTB. The causal relationship between systemic infections, such as pyelonephritis and pneumonia, followed by CAM and PTB has been investigated for a long time (Knox and Pratt 1990a). Additionally, in recent years, lower genital infections, such as those of the vagina and cervical canal, and their relationship with premature births have also been highlighted.

The following are the recent considerations on infection-induced PTB: (1) infection is often subclinical, (2) anaerobic bacteria and *Mycoplasma* are the infection-causing bacteria, and (3) the route of infection is from the external genitalia. It is mainly ascending, but occasionally hematogenous. PTB, especially that occurring at < 30 weeks, is assumed to involve infection in approximately 50% of cases (Lockwood 2002). Commensal and infective bacteria in the vagina are diverse and complicate the pathology of ascending infections (from bacterial vaginosis to cervicitis and CAM), which are considered to be the main pathogenesis of PTB.

As mentioned earlier, bacterial vaginosis is strongly associated with late miscarriage and has been reported to be one of the risk factors of premature birth. However, although a causal relationship is recognized, there is currently no fixed guideline for the management of bacterial vaginosis during pregnancy. Vaginal culture tests are performed to diagnose changes in the flora, but the pathogenicity of each is still unclear owing to the diverse species of microorganisms harbored in the vagina. Additionally, the causal relationship between the onset of PTB and CAM is well established (Hillier et al. 1995a, b). The induction of PTB by bacterial infection and inflammation has already been demonstrated in many human and animal studies. Cervicitis and bacterial vaginosis, which are considered as the precursors of CAM, infection, or inflammation caused by bacteria, cause premature membrane rupture and subsequent premature birth. According to a recent Ministry of Health, Labor and Welfare study (Organization 2017), the proportion of pregnant women diagnosed with bacterial vaginosis during pregnancy is increasing above the reported rate of 20% (Shimano et al. 2004).

As described above, there is a background surrounding the onset of premature birth. Interestingly, LF, a prebiotic that does not rely on conventional antibacterial agents, is attracting attention for its preventive effect on premature birth.

Recently, Ochoa and Sizonenko (2017) reviewed the efficacy of LF for improving the prognosis of neonates and preterm infants. The mechanisms by which LF contributes to the improvement of the prognosis of preterm infants have been shown to involve various effects, such as promotion of intestinal flora maturation, direct action on bacteria, inhibition of biofilm formation, downregulation of pro-inflammatory cytokines, and regulation of host immune response. Artym et al. (2021) reviewed the antimicrobial and prebiotic activity of LF in the female reproductive tract. Taking into consideration the problems surrounding the onset and prevention of premature birth, as described above, we reviewed the effects of LF in the prevention of premature birth, focusing on our own cases.

It was shown that human cervical epithelial cells produce LF, and its function is enhanced during pregnancy. In cases with cervicitis and CAM, the LF concentration in the cervical mucus decreased and the corresponding expression of LF in cervical epithelial cells decreased (Sawada et al. 2006a, b). It is possible that LF plays a role in the inflammatory defense mechanism in cervicitis tissue, probably because of it being prone to inflammation. In contrast, the high LF concentration in amniotic fluid in the CAM group was considered to be due to increased LF production in the fetus due to inflammation, suggesting its use as a potential biomarker. In cases with CAM, the LF concentration in neonatal saliva was high, suggesting that LF is also involved in the anti-inflammatory response in the fetus (Otsuki et al. 1997).

The antibacterial and anti-cytokine effects of rh-LF appeared only in mucus-producing cells among human cervical epithelial cells, suggesting the importance of cervical mucus-producing cells for the activity of rh-LF. In the presence of rh-LF, mucus-producing cervical epithelia may play an important role in restricting bacterial growth by helping rh-LF adhesion to bacteria (Johswich et al. 2013), since rh-LF controlled *E. coli* development in the mucus-producing cells (HeLa cell). In the presence of these cells, LF inhibited *E. coli* growth as well as LPS-induced cytokine production and activated macrophages (Sherman and Lönnerdal 2001). It was speculated that this antibacterial action of LF was not due to the opsonic effect of macrophages for bacterial phagocytosis (Otsuki et al. 2002a, b), but was mediated by the promotion of macrophage production by components, such as NO (Otsuki et al. 2000c).

The effect of LF administration was evaluated using PTB animal models. The results of the PTB suppression effect of LF in animals was evident and the effect was also shown to be mediated by the inhibition of cytokine production in the mother animal (Mitsuhashi et al. 2000) (Hasegawa et al. 2005a, b). These findings were also supported by pathological evaluations of the amniotic membrane and placenta. Additionally, in the *E. coli*-induced PTB model, the mortality rate of LF-receiving mothers due to direct *E. coli* infection, hypercytokine storms, or early onset of uterine contractions, was low, and pathological changes were suppressed (Yakuwa et al. 2007) (Nakayama et al. 2008a, b). This indicates that LF may improve the final prognosis of PTB infants as well as their inflammatory and high cytokine status due to its natural presence in humans (especially high in breast milk). Thus, the use of LF at high concentrations is expected to be highly beneficial in clinical practice and has minimum side effects.

The mechanism of improving vaginal bacterial flora by oral administration of LF has not been clarified. According to a recent report, it is speculated that oral LF administration may promote IgA secretion in the gastrointestinal tract and activate immune cells (Arciniega-Martínez et al. 2016), leading to an improvement in the bacterial flora in the gastrointestinal tract and vagina. LF administration increases systemic immunity and locally restores *Lactobacillus* species in patients with a high risk of PTB, refractory vaginitis, and repeated PTB mainly due to intrauterine infection. Furthermore, the administration of LF may be effective in suppressing PTB (Otsuki et al. 2014) (Otsuki and Imai 2017). Consistency, Elmenam and Farouk (2021) reported the preventive effect of bovine LF on premature birth, but they reported on cases with sterile inflammation, i.e., cervical insufficiency without inflammation. A major mechanism of PTB is inflammation, and our data are important in this regard.

Herein, LF was administered to humans as a health food; however, there are several restrictions and problems when it comes to the viewpoint and development of pharmaceuticals (Abad et al. 2021). The approval standards in Japan are even more stringent than those in the United States. Since LF has various functions, further rigorous research is desired in the future (Kilic et al. 2017a, b).

## Conclusion

LF may play a role in inflammatory protection in pregnant human cervical tissue. The antibacterial and anti-cytokine effects of LF in human-derived mucus-producing cervical cell lines have also been demonstrated. It was clarified that LF suppresses PTB and improves the prognosis of pups in inflammation-induced PTB animal models. Thus, based on the findings of this review, to our knowledge, we have identified the first ever clinical application of LF, a prebiotic contained in breast milk, for the purpose of suppressing PTB in humans. This strategy, applicable to high-risk cases of PTB, improves the vaginal flora by activating the systemic immune system; thus, it could be extremely effective in suppressing PTB. There are limited reports on LF and PTB prevention. However, it is clear that the causes of PTB and refractory vaginitis are associated with host immunity, and it may be useful to focus on the anti-inflammatory effect of LF. Further studies in this regard are expected in the future.

## Declarations

### Research involving human and animals rights

The protocol for animals were approved by the Animal Use and Care Committee of Showa University and the New Drug Development Research Center. We also obtained ethical approval from our Institutional Review Board and written informed consent from each patient.

## References

[CR1] Abad I, Conesa C, Sanchez L (2021). Development of encapsulation strategies and composite edible films to maintain lactoferrin bioactivity: a review. Materials (basel).

[CR3] Andrews WW, Hauth JC, Goldenberg RL, Gomez R, Romero R, Cassell GH (1995). Amniotic fluid interleukin-6: correlation with upper genital tract microbial colonization and gestational age in women delivered after spontaneous labor versus indicated delivery. Am J Obstet Gynecol.

[CR5] Arciniega-Martinez IM, Campos-Rodriguez R, Drago-Serrano ME, Sanchez-Torres LE, Cruz-Hernandez TR, Resendiz-Albor AA (2016). Modulatory effects of oral bovine lactoferrin on the iga response at inductor and effector sites of distal small intestine from BALB/c mice. Arch Immunol Ther Exp (warsz).

[CR6] Artym J, Zimecki M (2021). Antimicrobial and prebiotic activity of lactoferrin in the female reproductive tract: a comprehensive review. Biomedicines.

[CR7] Beck S, Wojdyla D, Say L, Betran AP, Merialdi M, Requejo JH, Rubens C, Menon R, Van Look PF (2010). The worldwide incidence of preterm birth: a systematic review of maternal mortality and morbidity. Bull World Health Organ.

[CR8] Bennett PR, Elder MG (1992). The mechanisms of preterm labor: common genital tract pathogens do not metabolize arachidonic acid to prostaglandins or to other eicosanoids. Am J Obstet Gynecol.

[CR9] Bick D (2012). Born too soon: the global issue of preterm birth. Midwifery.

[CR10] Cox SM, MacDonald PC, Casey ML (1988). Assay of bacterial endotoxin (lipopolysaccharide) in human amniotic fluid: potential usefulness in diagnosis and management of preterm labor. Am J Obstet Gynecol.

[CR11] Elmenam HS, Farouk MH (2021). Bovine lactoferrin in preterm labor with sterile inflammation. Sci J Al-Azhar Med Faculty Girls.

[CR521] Goldenberg RL, Hauth JC, Andrews WW (2000). ntrauterine infection and preterm delivery. N Engl J Med.

[CR12] Hasegawa A, Otsuki K, Sasaki Y, Sawada M, Mitsukawa K, Chiba H, Nagatsuka M, Okai T, Kato A (2005). Preventive effect of recombinant human lactoferrin in a rabbit preterm delivery model. Am J Obstet Gynecol.

[CR13] Hillier SL, Nugent RP, Eschenbach DA, Krohn MA, Gibbs RS, Martin DH, Cotch MF, Edelman R, Pastorek JG, Rao AV (1995). Association between bacterial vaginosis and preterm delivery of a low-birth-weight infant. The Vaginal Infections and prematurity Study Group. N Engl J Med.

[CR522] Johswich KO, McCaw SE, Islam E, Sintsova A, Gu A, Shively JE, Gray-Owen SD (2003). In vivo adaptation and persistence of Neisseria meningitidis within the nasopharyngeal mucosa. PLoS Pathog.

[CR15] Keelan JA, Blumenstein M, Helliwell RJ, Sato TA, Marvin KW, Mitchell MD (2003). Cytokines, prostaglandins and parturition–a review. Placenta.

[CR17] Kilic E, Novoselova MV, Lim SH, Pyataev NA, Pinyaev SI, Kulikov OA, Sindeeva OA, Mayorova OA, Murney R, Antipina MN, Haigh B, Sukhorukov GB, Kiryukhin MV (2017). Formulation for oral delivery of lactoferrin based on bovine serum albumin and tannic acid multilayer microcapsules. Sci Rep.

[CR18] Knox JR, Pratt RF (1990). Different modes of vancomycin and D-alanyl-D-alanine peptidase binding to cell wall peptide and a possible role for the vancomycin resistance protein. Antimicrob Agents Chemother.

[CR19] Lima MF, Kierszenbaum F (1985). Lactoferrin effects on phagocytic cell function. I. Increased uptake and killing of an intracellular parasite by murine macrophages and human monocytes. J Immunol.

[CR20] Lima MF, Kierszenbaum F (1987). Lactoferrin effects of phagocytic cell function. II. The presence of iron is required for the lactoferrin molecule to stimulate intracellular killing by macrophages but not to enhance the uptake of particles and microorganisms. J Immunol.

[CR21] Lockwood CJ (2002). Predicting premature delivery–no easy task. N Engl J Med.

[CR22] MacDorman MF, Matthews TJ, Mohangoo AD, Zeitlin J (2014). International comparisons of infant mortality and related factors: United States and Europe, 2010. Natl Vital Stat Rep.

[CR23] Mastromarino P, Vitali B, Mosca L (2013). Bacterial vaginosis: a review on clinical trials with probiotics. New Microbiol.

[CR24] Mathews TJ, Driscoll AK (2017). Trends in infant mortality in the United States, 2005–2014. NCHS Data Brief.

[CR25] McGregor JA, French JI, Jones W, Milligan K, McKinney PJ, Patterson E, Parker R (1994). Bacterial vaginosis is associated with prematurity and vaginal fluid mucinase and sialidase: results of a controlled trial of topical clindamycin cream. Am J Obstet Gynecol.

[CR27] Mitsuhashi Y, Otsuki K, Yoda A, Shimizu Y, Saito H, Yanaihara T (2000). Effect of lactoferrin on lipopolysaccharide (LPS) induced preterm delivery in mice. Acta Obstet Gynecol Scand.

[CR28] Miyazawa K, Mantel C, Lu L, Morrison DC, Broxmeyer HE (1991). Lactoferrin-lipopolysaccharide interactions. Effect on lactoferrin binding to monocyte/macrophage-differentiated HL-60 cells. J Immunol.

[CR29] Nakayama K, Otsuki K, Yakuwa K, Hasegawa A, Sawada M, Mitsukawa K, Chiba H, Nagatsuka M, Okai T (2008). Recombinant human lactoferrin inhibits matrix metalloproteinase (MMP-2, MMP-3, and MMP-9) activity in a rabbit preterm delivery model. J Obstet Gynaecol Res.

[CR523] Ochoa TJ, Sizonenko SV (2017). Lactoferrin and prematurity: a promising milk protein?. Biochem Cell Biol.

[CR30] Ono T, Murakoshi M, Suzuki N, Iida N, Ohdera M, Iigo M, Yoshida T, Sugiyama K, Nishino H (2010). Potent anti-obesity effect of enteric-coated lactoferrin: decrease in visceral fat accumulation in Japanese men and women with abdominal obesity after 8-week administration of enteric-coated lactoferrin tablets. Br J Nutr.

[CR31] Organization MCH (2017) Matern Child Health Stat Jpn Mothers & Childrens’ Health & Welfare Association

[CR33] Otsuki K, Imai N (2017). Effects of lactoferrin in 6 patients with refractory bacterial vaginosis. Biochem Cell Biol.

[CR38] Otsuki K, Yoda A, Hirose K, Shimizu Y, Saito H, Takumi Y (1997). Salivary lactoferrin in neonates with chorioamnionitis. Showa Univ J Med Sci.

[CR39] Otsuki K, Yoda A, Toma Y, Shimizu Y, Saito H, Yanaihara T (1998). Lactoferrin and interleukin-6 interaction in amniotic infection. Adv Exp Med Biol.

[CR40] Otsuki K, Yoda A, Saito H, Mitsuhashi Y, Toma Y, Shimizu Y, Yanaihara T (1999). Amniotic fluid lactoferrin in intrauterine infection. Placenta.

[CR42] Otsuki K, Lonnerdal B, Sherman MP (2002). Bacterial growth on human cervical epithelia: a new assay for testing killing by lactoferrin. Biochem Cell Biol.

[CR43] Otsuki K, Lonnerdal B, Sherman MP (2002). Bacterial opsonin for macrophages?. Biochem Cell Biol.

[CR41] Otsuki K, Lonnerdal B, Sherman MP (2000). Is lactoferrin a sauce that promotes ingestion of E. coli by lung macrophages?. FASEB J.

[CR44] Otsuki K, Yakuwa K, Sawada M, Hasegawa A, Sasaki Y, Mitsukawa K, Chiba H, Nagatsuka M, Saito H, Okai T (2005). Recombinant human lactoferrin has preventive effects on lipopolysaccharide-induced preterm delivery and production of inflammatory cytokines in mice. J Perinat Med.

[CR45] Otsuki K, Tokunaka M, Oba T, Nakamura M, Shirato N, Okai T (2014). Administration of oral and vaginal prebiotic lactoferrin for a woman with a refractory vaginitis recurring preterm delivery: appearance of lactobacillus in vaginal flora followed by term delivery. J Obstet Gynaecol Res.

[CR46] Sasaki Y, Otsuki K, Hasegawa A, Sawada M, Chiba H, Negishi M, Nagatsuka M, Okai T (2004). Preventive effect of recombinant human lactoferrin on lipopolysaccharide-induced preterm delivery in mice. Acta Obstet Gynecol Scand.

[CR47] Sawada M, Otsuki K, Mitsukawa K, Yakuwa K, Nagatsuka M, Okai T (2006). Cervical inflammatory cytokines and other markers in the cervical mucus of pregnant women with lower genital tract infection. Int J Gynaecol Obstet.

[CR48] Shau H, Kim A, Golub SH (1992). Modulation of natural killer and lymphokine-activated killer cell cytotoxicity by lactoferrin. J Leukoc Biol.

[CR49] Sherman MP, Lönnerdal BL (2001). Preventing *Escherichia* coli growth on cervical epithelium. Pediatr Res.

[CR50] Shimano S, Nishikawa A, Sonoda T, Kudo R (2004). Analysis of the prevalence of bacterial vaginosis and Chlamydia trachomatis infection in 6083 pregnant women at a hospital in Otaru, Japan. J Obstet Gynaecol Res.

[CR51] Shimizu H (2004). Development of an enteric-coated lactoferrin tablet and its application. Biometals.

[CR52] Taylor-Robinson D, Furr PM (1997). Genital mycoplasma infections. Wien Klin Wochenschr.

[CR53] Yakuwa K, Otsuki K, Nakayama K, Hasegawa A, Sawada M, Mitsukawa K, Chiba H, Nagatsuka M, Okai T (2007). Recombinant human lactoferrin has a potential to suppresses uterine cervical ripening in preterm delivery in animal model. Arch Gynecol Obstet.

[CR54] Zhang GH, Mann DM, Tsai CM (1999). Neutralization of endotoxin in vitro and in vivo by a human lactoferrin-derived peptide. Infect Immun.

[CR55] Zimecki M, Wlaszczyk A, Cheneau P, Brunel AS, Mazurier J, Spik G, Kubler A (1998). Immunoregulatory effects of a nutritional preparation containing bovine lactoferrin taken orally by healthy individuals. Arch Immunol Ther Exp (warsz).

